# Impact of Neutrophil-to-Lymphocyte Ratio on Stroke Severity and Clinical Outcome in Anterior Circulation Large Vessel Occlusion Stroke

**DOI:** 10.3390/diagnostics14242880

**Published:** 2024-12-21

**Authors:** Zülfikar Memiş, Erdem Gürkaş, Atilla Özcan Özdemir, Bilgehan Atılgan Acar, Muhammed Nur Ögün, Emrah Aytaç, Çetin Kürşad Akpınar, Eşref Akıl, Murat Çabalar, Ayça Özkul, Ümit Görgülü, Hasan Bayındır, Zaur Mehdiyev, Şennur Delibaş Katı, Recep Baydemir, Ahmet Yabalak, Ayşenur Önalan, Türkan Acar, Özlem Aykaç, Zehra Uysal Kocabaş, Serhan Yıldırım, Hasan Doğan, Mehmet Semih Arı, Mustafa Çetiner, Ferhat Balgetir, Fettah Eren, Alper Eren, Nazım Kızıldağ, Utku Cenikli, Aysel Büşra Şişman Bayar, Ebru Temel, Alihan Abdullah Akbaş, Emine Saygın Uysal, Hamza Gültekin, Cebrail Durmaz, Sena Boncuk Ulaş, Talip Asil

**Affiliations:** 1 Department of Neurology, Haseki Training and Research Hospital, University of Health Sciences, Istanbul 34130, Turkey; zulfikarmemis1@gmail.com (Z.M.); tmlebru@outlook.com (E.T.); 2 Department of Neurology, Kartal Dr. Lütfi Kırdar City Hospital, University of Health Sciences, Istanbul 34130, Turkey; erdemgurkas@yahoo.com (E.G.); draysenurkaymaz@gmail.com (A.Ö.); 3 Department of Neurology, Faculty of Medicine, Eskişehir Osmangazi University, Eskişehir 26040, Turkey; atillaozcanozdemir@gmail.com (A.Ö.Ö.); drzlm@yahoo.com (Ö.A.); drzuysal@hotmail.com (Z.U.K.); 4 Department of Neurology, Faculty of Medicine, Sakarya University, Sakarya 54100, Turkey; tdeniz38@hotmail.com; 5 Department of Neurology, Faculty of Medicine, Bolu Abant İzzet Baysal University, Bolu 14030, Turkey; dr.mogun@gmail.com; 6 Department of Neurology, Faculty of Medicine, Fırat University, Elazig 23119, Turkey; dremrah_aytac@hotmail.com (E.A.); ferhatbalgetir@hotmail.com (F.B.); 7 Department of Neurology, Faculty of Medicine, Samsun University, Samsun 55080, Turkey; dr_ckakpinar@hotmail.com (Ç.K.A.); dr.hasandogan@outlook.com (H.D.); 8 Department of Neurology, Faculty of Medicine, Dicle University, Diyarbakir 21280, Turkey; esrefakil@gmail.com (E.A.); hamzagultekin72@hotmail.com (H.G.); dr.cdurmaz@gmail.com (C.D.); 9 Department of Neurology, İstanbul Başakşehir Çam Sakura City Hospital, Istanbul 34480, Turkey; mcabalar@gmail.com (M.Ç.); ozkulayca@hotmail.com (A.Ö.); semih-ari@hotmail.com (M.S.A.); 10 Department of Neurology, Ankara Bilkent City Hospital, Ankara 06800, Turkey; drumitgorgulu@hotmail.com (Ü.G.); h.bayindir@hotmail.com (H.B.); 11 Department of Neurology, Ankara Etlik City Hospital, Ankara 06710, Turkey; dr_mehdiyev@yahoo.com; 12 Department of Neurology, Antalya Training and Research Hospital, Antalya 07070, Turkey; sennurdelibas@yahoo.com; 13 Department of Neurology, Faculty of Medicine, Erciyes University, Kayseri 38030, Turkey; recep.baydemir@gmail.com; 14 Department of Neurology, Faculty of Medicine, Düzce University, Duzce 81620, Turkey; yabalakahmet@gmail.com; 15 Department of Neurology, Kocaeli City Hospital, Kocaeli 41060, Turkey; serhan_yildirim@yahoo.com; 16 Department of Neurology, Faculty of Medicine, Kutahya Health Sciences University, Kütahya 43020, Turkey; drcetiner76@gmail.com (M.Ç.); sayginemine43@gmail.com (E.S.U.); 17 Department of Neurology, Faculty of Medicine, Selçuk University, Konya 42130, Turkey; dreren42@hotmail.com; 18 Department of Neurology, Faculty of Medicine, Atatürk University, Erzurum 25240, Turkey; alpereren25@gmail.com (A.E.); kizildag.noro@gmail.com (N.K.); 19 Department of Neurology, Faculty of Medicine, Muğla Sıtkı Koçman University, Muğla 48000, Turkey; utkucenikli@yahoo.com; 20 Silvan Dr. Yusuf Azizoğlu State Hospital, Diyarbakır 21640, Turkey; busrasisman@gmail.com; 21 Department of Neurology, Sakarya University Training and Research Hospital, Sakarya 54100, Turkey; alihanakbas97@gmail.com; 22 Independent Researcher, Sakarya 54100, Turkey; senaboncuk@gmail.com; 23 Department of Neurology, Faculty of Medicine, Biruni University, Istanbul 34015, Turkey; talip_asil@yahoo.com

**Keywords:** stroke, neutrophil–lymphocyte ratio, inflammation, prognostic biomarkers, mechanical thrombectomy

## Abstract

**Background:** The prognostic value of the neutrophil–lymphocyte ratio (NLR) in ischemic stroke remains debated due to cohort variability and treatment heterogeneity across studies. This study evaluates the relationship between admission NLR, stroke severity and 90-day outcomes in patients with anterior circulation large vessel occlusion (LVO) undergoing early, successful revascularization. **Methods:** A retrospective multicenter study was conducted with 1082 patients treated with mechanical thrombectomy for acute ischemic stroke. The relationship between admission NLR, baseline National Institutes of Health Stroke Scale (NIHSS), 24 h NIHSS and 90-day modified Rankin Scale (mRS) outcomes was analyzed using logistic regression. **Results:** Admission NLR correlated weakly but significantly with both baseline (*p* = 0.018) and 24 h (*p* = 0.005) NIHSS scores, reflecting stroke severity. However, multivariate analysis showed that higher 24 h NIHSS scores (OR 0.831, *p* = 0.000) and prolonged puncture-to-recanalization times (OR 0.981, *p* = 0.000) were independent predictors of poor 90-day outcomes, whereas NLR was not (*p* = 0.557). **Conclusions:** Admission NLR is associated with stroke severity but does not independently predict clinical outcomes at 90 days in patients achieving early and successful revascularization. These findings underscore the critical role of inflammation in the acute phase of stroke but suggest that its prognostic value for long-term outcomes is limited in this context.

## 1. Introduction

Ischemic stroke is considered to be the second most common cause of death and the most common cause of morbidity. Therefore, its treatment and long-term follow-up are of critical importance for the patient. Although various treatment options exist, the primary goal remains the restoration of reperfusion. The treatments are thrombolytic therapy and thrombectomy for suitable patients. Despite appropriate treatment, more than half of patients remain seriously disabled or die [1,2]. According to a 2023 study, the incidence of ischemic stroke in Turkey is 93.2–108.6 per 100,000 and the total stroke incidence is 141.7–158 per 100,000 [3]. According to 2019 data, the most common cause of death in Turkey is cardiovascular diseases (36.8%); stroke ranks second in this subgroup, after coronary syndromes, and stroke-related deaths have increased by 56% since 2002 [3]. The etiology of ischemic stroke in 40% of patients occurs secondary to large vessel occlusion [4]. There are no clear data on the cause of this occlusion and the content of the thrombus that forms. In 2017, Brinjikji et al. examined the histopathological features of the thrombi and looked at the platelet, leukocyte and erythrocyte ratios and thought that the ratios varied depending on the source of the thrombus [5]. Recent studies suggest that inflammation plays an important role in thrombus formation and that neutrophils are the most responsible for this [6]. Neutrophil extracellular traps (NETs) are released by neutrophils, initiate pro-inflammatory processes and cause arterial and venous thrombogenesis [7,8].

Although it has been stated that inflammation is important in thrombus formation, anti-inflammatory therapies remain ineffective for ischemic stroke. This has led researchers to question whether inflammatory markers can be used in the prognostic evaluation of patients. Neutrophil–lymphocyte ratio (NLR), which has recently been frequently used as an inflammatory biomarker, combines the destructive effect of neutrophils and the anti-inflammatory effect of lymphocytes and reflects the interplay between neutrophilic inflammation and lymphocyte-mediated immunity, offering insights into systemic inflammation [9].

After the appearance of acute ischemic stroke (AIS), neutrophils and lymphocytes are involved in the pathophysiological process. Neutrophils migrate to vascular lesions, release proteases and free oxygen radicals, and directly or indirectly aggravate brain damage [10]. Neutrophils can also increase the expression of matrix metalloproteinase-9 and directly destroy the blood–brain barrier (BBB), resulting in secondary brain damage or hemorrhagic transformation (HT) [11].

On the other hand, lymphocytes also play an important role in the development of inflammation. The accumulation of lymphocytes in the damaged vascular endothelium causes the progression of the disease. In the advanced stages of the disease, lymphocytes play a protective role, mainly as anti-inflammatory actors [11]. In addition, neutrophils are the first cells to cross the BBB and initiate damage within the first hour after stroke. Other lymphocytes then follow, increasing the damage to the BBB. In this context, NLR gains importance both as an easily accessible blood-based biomarker and because it is involved in the pathogenesis of stroke from the first stages of damage to the BBB [12].

NLR reflects the combined roles of neutrophils and lymphocytes, indicating the body’s inflammatory and stress status [13]. NLR, which shows the balance between innate immunity and acquired immunity, has been evaluated in terms of prognosis prediction in sepsis, cancer patients and cardiac events, and significant results have been obtained [14,15,16].

Although there are studies showing that NLR can be used in short-term prognosis prediction of ischemic stroke, there are still no clear data [17,18,19]. Our aim in this study is to investigate the effect of NLR on stroke severity and prognosis in AIS patients who underwent successful revascularization in the early period.

## 2. Materials and Methods

### 2.1. Study Population

This is a multicenter, retrospective study conducted with the participation of 19 stroke centers in Turkey, including the prospective and consecutively collected data of 1289 adult patients with anterior circulation AIS between January 2021 and December 2022. After applying the exclusion criteria and using consecutive patient sampling to reduce selection bias, 1082 patients who achieved successful revascularization were included in the study.

All patients in the study population met standard acute stroke treatment criteria and underwent mechanical thrombectomy in accordance with current stroke guidelines. The decision to perform thrombectomy was based on the identification of large vessel occlusion via computed tomography angiography in patients presenting within 6 h of estimated onset.

**Figure 1:**
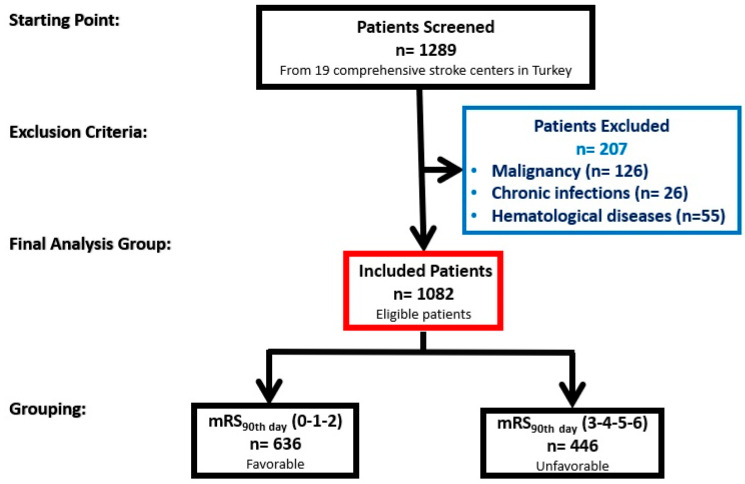
Patient selection flowchart.

Exclusion criteria were as follows: age < 18 or >80 years, modified Rankin Scale (mRS) > 2, brain hemorrhage, intracerebral mass or computed tomography diagnosis of cerebrovascular injury due to trauma, systemic diseases that may cause changes in complete blood count, autoimmune diseases, hematological diseases, chronic infections or using immunosuppressive therapy, posterior circulation large vessel occlusion or internal carotid artery (ICA) tandem occlusion. Patients over 80 years of age and those with a baseline mRS > 2 were excluded to minimize potential confounding factors. Advanced age and significant pre-stroke disability can independently influence clinical outcomes, frailty and systemic inflammation, thereby introducing bias in the analysis of NLR and stroke outcomes. Patients were categorized based on their mRS score at 90 days (mRS 0,1,2; good clinical outcome, mRS 3,4,5,6; poor clinical outcome) (Figure 1). Stroke severity at baseline was determined using the admission National Institutes of Health Stroke Scale (NIHSS) score.

A total of 1289 potentially eligible participants were recruited from 19 comprehensive stroke centers in Turkey. After excluding 207 patients (malignancy: *n* = 126, chronic infections: *n* = 26 and hematological diseases: *n* = 55), 1082 patients were included in the study. The included patients were categorized based on their modified Rankin Scale (mRS) scores on the 90th day. Patients with mRS scores of 0-1-2 (favorable outcomes) accounted for 636 participants, while those with mRS scores of 3-4-5-6 (unfavorable outcomes) totaled 446 participants.

The primary outcome was poor functional outcome at 90 days reflected by an mRS score of 3 to 6. Successful revascularization was defined as a post-treatment modified treatment in cerebral infarction (mTICI) score of 2b to 3.

Written consent was obtained from patients or their relatives (for aphasic or unconscious patients) for participation in the study, and the study was approved by the ethics committee of Haseki Training and Research Hospital.

### 2.2. Patient Data Evaluation and Analysis

We collected patient data including demographic characteristics, clinical data and laboratory parameters. Patient characteristics included age, gender, smoking status, alcohol consumption, hypertension, diabetes, atrial fibrillation (AF), asthma, hyperlipidemia (HL) and coronary heart disease (CHD). Clinical data included admission systolic blood pressure (BP), admission diastolic BP, admission NIHSS score, symptom–puncture time (time from onset to inguinal puncture), puncture–recanalization time (time from inguinal puncture to reperfusion) and Alberta Stroke Program Early Computed Tomography score (ASPECTS).

Laboratory parameters were blood glucose (BG), lymphocyte count, neutrophil count, red blood cell distribution width (RDW), platelet (PLT) count, NLR, platelet–lymphocyte ratio (PLR). All peripheral venous blood tests were performed before thrombectomy. Laboratory parameters were determined by optical laser scatter and fluorescent staining methods using Mindray BC6200 (Shenzhen Mindray Bio-Medical Electronics Co., Ltd., Shenzhen, China), Sysmex XN-1000 (Sysmex Corporation, Kobe, Japan) and/or Celltac G MEK-9100 (Nihon Kohden Corporation, Tokyo, Japan) devices. NLR was calculated by dividing the neutrophil count by the lymphocyte count. PLR was calculated by dividing the platelet count (10^9^/L) by the lymphocyte count.

### 2.3. Statistics

The Kolmogorov–Simirnov test was used to examine whether continuous quantitative variables showed normal distribution, and, at the same time, the skewness and kurtosis coefficients of the variables were examined. While *n* and percentage were reported for categorical variables, minimum, maximum, median and mean ± standard deviation (SD) were reported for continuous variables. When examining the relationships between variables, the correlations for variables that did not show normal distribution (neutrophil, lymphocyte, platelets, NLR, PLR) were calculated using Spearman’s rho; Pearson’s product-moment correlation coefficient was calculated for normally distributed variables (24th hour NIHSS score, admission NIHSS score).

The skewness coefficient of variables with non-normal distribution ranged between 2.09 and 32.10, while the skewness coefficient of variables with normal distribution was in the range of 0.12–0.92. Relationships between categorical variables were examined using chi-square analysis. Fisher’s exact test was used for those with an expected value below 5. Logistic regression analysis was performed to estimate the dichotomized mRS score (mRS scores of 0, 1 and 2 as zero and those of 3, 4, 5 and 6 as one). As a result of both single and multiple logistic regression analysis, 95% confidence intervals and likelihood ratios were calculated. NLR, 24th hour NIHSS score and Neutrophil variables were not considered together in multiple logistic regression because the variables could affect each other. All analyses were performed using SPSS 26 software with a significance level of *p* < 0.05.

## 3. Results

Of the 1082 patients, 545 (50.4%) were female and 537 (49.6%) were male, with a mean age of 65.1 ± 12.2 years. The mean NLR was 5.3 ± 6.3. The time between the onset of symptoms and inguinal puncture was 193 ± 85.7 min (range 9–360). The mean NIHSS score on admission was 14.81 ± 4.8, with a range of 6–32 and a median of 15. A total of 636 (58.7%) patients had good functional outcomes 3 months after acute ischemic stroke. Of the total cohort, 914 patients (80%) had M1 occlusion, M2 occlusion occurred in 164 patients (14.3%), carotid T or L occlusion in 63 patients (5.5%), and anterior cerebral artery A1 occlusion in 2 patients (0.2%). Table 1 summarizes the basic demographic, laboratory, and clinical data of the patients.

In the correlation analysis of inflammatory parameters and admission NIHSS, neutrophil values and NLR values showed a statistically significant, weak positive correlation with admission NIHSS score. (*p* = 0.003, rho = 0.131, *p* = 0.018, rho = 0.143, respectively).

Similarly, in the correlation analysis of inflammatory parameters with 24th hour NIHSS score, neutrophil value and NLR values showed a statistically significant, weak positive correlation with 24th hour NIHSS score (*p* = 0.000, rho = 0.152, *p* = 0.005, rho = 0.186, respectively) (Table 2).

Of the 1082 patients, 446 (41.3%) had unfavorable 90-day functional outcomes. The favorable group had a lower proportion of males and younger individuals than the unfavorable group. The differences between the two groups are detailed in Table 3. Hypertension and diabetes were more prevalent in the unfavorable outcome group (*p* value 0.003 and 0.001, respectively). Patients in the unfavorable outcome group also had higher admission NIHSS scores (*p* = 0.001), higher neutrophil counts (*p* = 0.001), higher NLR values (*p* = 0.013), longer puncture–revascularization time (*p* = 0.001) and lower first pass revascularization rates (*p* = 0.002) (Table 3, Supplementary Table S1, Supplementary Figure S1).

Univariate logistic regression analyses and multivariate logistic regression analyses performed after adjusting for confounding variables revealed no independent association between any of the inflammatory parameters and 90th day functional outcomes. It was observed that the high 24th hour NIHSS score (OR, 0.831; 95% CI, 0.803–0.859; *p* = 0.000) and the length of puncture–revascularization time (OR, 0.981; 95% CI, 0.973–0.989; *p* = 0.000) were independent predictors of poor clinical outcome on the 90th day. In univariate logistic regression analysis, it was seen that high 24th hour NIHSS score (OR, 1.242; 95% CI, 1.209–1.277; *p* = 0.000), long puncture–revascularization time (OR, 1.013; 95% CI, 1.007–1.018; *p* = 0.000) and first pass recanalization (OR, 0.675; 95% CI, 0.528–0.863; *p* = 0.591) were independent predictors in favor of poor clinical outcomes on the 90th day (Table 4, Supplementary Table S2).

**Figure 2:**
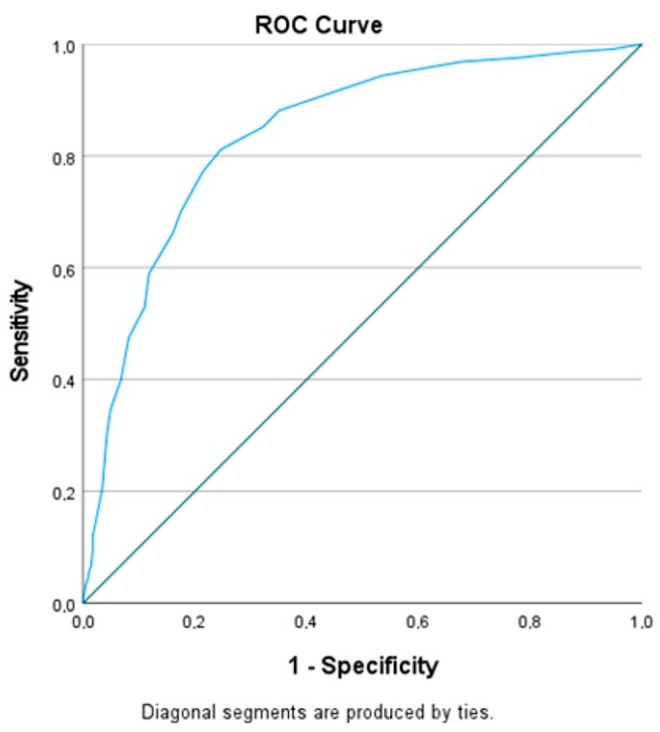
ROC curve for 24th hour NIHSS score predicting poor 90-day outcomes (AUC = 0.84, *p* < 0.001).

Receiver Operating Characteristic (ROC) curve analysis was performed for the significant variables. Variables with an area under curve (AUC) value above 0.60 were analyzed. The ROC curve for 24th hour NIHSS score was found to be highly associated with predicting poor 90-day outcomes, with an AUC of 0.84 (Figure 2, Table 5).

## 4. Discussion

In this study, firstly, the predictive value of NLR levels at admission in AIS patients for stroke severity was investigated. The results showed that high NLR levels were blunted and statistically associated with the elevation of admission NIHSS, which indicates stroke severity. In addition, we observed that a high 24th hour NIHSS score, which can be considered as an indicator of early neurological deterioration, and high admission-NLR levels were blunted and statistically associated. Many studies show that NLR is closely related to cerebrovascular diseases. A large meta-analysis of data from 9563 patients by Wang et al. showed that high NLR predicts short-term adverse outcomes in AIS patients [20]. Another review study showed that NLRs were higher in patients with unfavorable prognosis in hemorrhagic strokes and that this could be a predictive marker in the short term [21]. In a multicenter study by Piri Cinar B et al., which included 737 patients, high NLR levels were associated with high admission-NIHSS [22]. Aytaç E et al., in their study with 209 patients, showed that high admission-NLR values were associated with high admission-NIHS scores [23]. In a recent meta-analysis of nine studies, high NLR values were found to be associated with high NIHSS scores and early neurological deterioration [24]. Our study’s data, which demonstrate the relationship between baseline NLR values and NIHSS scores at admission and 24 h, align with findings in the literature.

To our knowledge, this is the first study to evaluate the relationship between NLR levels and 90-day functional outcome in patients with a symptom–puncture time within the first 6 h and successful anterior circulation revascularization. We focused on the NLR values in this patient group for several important reasons. First, most of the previous studies have assessed inflammatory responses in all ischemic stroke patients, regardless of revascularization success [25,26]. Second, considering that mechanical thrombectomy (MT) can be performed up to 24 h, symptom–puncture times vary greatly across studies in the literature [22,23,24,27,28]. Finally, most studies evaluated ischemic stroke patients in the same pool without making any distinction between the anterior and posterior systems [24,29,30]. We thought that this study, which was conducted in a specific patient group with a higher number of patients, could provide more valuable data. At the end of our study, the admission NLR value was not associated with the 3rd month mRS score. In contrast to our study, a recent prospective study of 341 ischemic stroke patients found that high NLR levels were associated with poor clinical outcome in the 3rd month. However, only those over 50 years of age and those with a baseline NIHSS score below 10 were included in that study [26]. In a meta-analysis conducted in 2022 evaluating PLR in AIS patients treated with reperfusion therapy, data of 4788 patients were analyzed and it was shown that low admission-PLR values were associated with early neurologic recovery and good functional outcome on day 90. This meta-analysis emphasizes the importance of inflammatory parameters other than NLR in AIS and is inconsistent with our results in terms of the association with 90th day clinical outcome [31]. In another meta-analysis study of 10,308 patients examining the role of NLR in AIS patients undergoing reperfusion therapy, higher admission or delayed NLR are significantly associated with worse morbidity, mortality and safety outcomes in AIS patients [32]. Unlike these meta-analyses, the lack of association between outcome and NLR in our study may have resulted from methodological differences such as the inclusion of successful recanalization patients and the analysis of pre-procedural NLR values.

In another study conducted by Chen et al. on AIS patients receiving MT and/or intravenous thrombolytic therapy (IVTPA), it was shown that both baseline NLR and first-day NLR values were high, reducing functional independence. However, it has been emphasized that the first-day NLR value has a stronger relationship [30]. In studies evaluating patients who underwent successful revascularization, the focus has generally been on the NLR values studied after revascularization rather than the admission NLR values. Li S et al. studied 237 cases who were admitted within the first 12 h and had successful revascularization; NLR was calculated for each day in the first 3 days of treatment and its relationship with 1st month mortality was examined. All three days’ NLR values were found to be associated with 1st month mortality [33]. In a study performed by Zou F et al., which included 160 ischemic stroke patients who underwent successful revascularization, high NLR values in the early period after revascularization were associated with poor prognosis at 3 months [34].

In another study of patients with anterior system stroke who were successfully revascularized, the admission NLR value was not associated with the 3rd month mRS score, but a significant relationship was found between the NLR value studied on the 1st day after revascularization and the 3rd month mRS score [28]. Similar to our study, in this study where patients who had successful revascularization were included in the study, the average symptom–recanalization time was calculated as 438 min. In our study, the average symptom–recanalization time was 239 min. Considering this situation, it is thought that the majority of the patients in this study were revascularized late, that the inflammatory process may have already started in these patients and that this situation may have affected the study results in terms of recanalization benefit.

In a study by Li SJ et al., there was no relationship found between the admission NLR value and neurological outcomes, but the post-MT NLR value was found to be predictive of neurological outcomes. Subgroup analyses of NLR assessment showed that patients with MT, successful revascularization, and higher inflammatory biomarkers did not have an increased risk of poor 3-month functional outcome; this result is consistent with the results in our study [25]. The relationship between inflammation and acute ischemic stroke is currently a focus of attention. In our study, we focused on one of the most common markers of inflammation, NLR, and ultimately demonstrated that NLR is not associated with poor prognosis after stroke in the setting of successful and timely revascularization. Given that NLR is increased by many factors, such as cardiovascular disease [35], chronic inflammatory diseases [36], systemic infections [37], and post-stroke pneumonia [38], this value may not be an independent predictor of clinical outcome when beneficial reperfusion is achieved, which is the primary goal in ischemic stroke.

This study has several limitations but also notable strengths, such as including a large cohort of patients who underwent early and successful revascularization. First, NLR values were studied only from venous blood taken at presentation. Considering the variability of inflammation over time, studying NLR values after MT or in the following days may yield different results. Secondly, although we listed the patient group that we selected as an advantage (early and successful revascularization) and included a more specific patient group, an analysis was made that still included too many variables affecting the mRS result. It is possible to obtain more valuable results by reducing the variables of the study group. Another limitation of our study is that infections or other systemic inflammatory responses after stroke were not analyzed. These data may provide important information about stroke when monitored over time. In addition, successful revascularization may reduce inflammation and affect NLR and other inflammatory parameter outcomes. However, we analyzed the NLR values before the procedure, when the patient was first admitted to the hospital, and the aim of our study was to examine the association of these values with stroke severity and clinical outcome. Variability in systemic inflammatory responses (e.g., pneumonia after stroke) may affect the prognostic value of NLR. Future studies may plan study designs with repeated NLR measurements after MT to address this limitation.

## 5. Conclusions

This study highlights a significant relationship between admission NLR and stroke severity in patients with anterior circulation large vessel occlusion undergoing early, successful revascularization. However, NLR was not found to be an independent predictor of long-term functional outcomes at 90 days. These results suggest that inflammation, as measured by NLR, is a determinant of acute stroke severity but not of long-term recovery in the context of effective and timely reperfusion therapy.

The findings of this study should be interpreted in the context of its design, which included only patients with early and successful revascularization and analyzed pre-procedural NLR values. Post-stroke inflammatory changes and delayed NLR measurements were not evaluated, which may limit the generalizability of the results. Future research should consider incorporating serial NLR measurements, broader patient cohorts, and additional inflammatory biomarkers to comprehensively assess their prognostic potential.

## Figures and Tables

**Table 1:** Patient characteristics, clinical findings and inflammatory parameters.

		Min–Max	Mean ± SD	*n*-%
Ages		19–80	65.1 ± 12.2	1082
Gender	Female			545–50.4%
	Male			537–49.6%
Body-Mass Index	Normal			784–72.5%
	Obesity			267–24.7%
	Cachectic			31–2.9%
Clinical Characteristics				
Hypertension	Yes			426–39.4%
	No			656–60.6%
Diabetes	No			725–67.0%
	Yes			357–33.0%
Coronary Artery Disease	No			692–64.0%
	Yes			390–36.0%
Congestive Heart Failure	No			905–83.6%
	Yes			177–16.4%
Asthma and/or COPD	No			964–89.1%
	Yes			118–10.9%
Atrial Fibrillation	No			640–59.1%
	Yes			442–40.9%
Alcohol Consumption	No			996–92.1%
	Yes			86–7.9%
Smoker	No			788–72.8%
	Yes			294–27.2%
Previous Stroke History	No			934–86.3%
	Yes			148–13.7%
Hyperlipidemia	No			515–47.6%
	Yes			567–52.4%
Inflammatory Parameters				
Neutrophil (10⁹/L)		0.1–112.18	8.05 ± 8.6	1082 (%100)
Lymphocyte (10⁹/L)		0.2–51.7	2.2 ± 2.6	1082 (%100)
PLT (10⁹/L)		10–996	236.6 ± 88.2	1082 (%100)
RDW (%)		9.5–172	14.8 ± 5.8	1082 (%100)
NLR		0.02–124.45	5.3 ± 6.3	1082 (%100)
PLR		3.33–193.0	155.9 ± 123.5	1082 (%100)
Admission NIHSS		4–32	14.81 ± 4.8	1082 (%100)
Procedural Data				
Symptom-to-Inguinal Puncture Time (min)		9–360	193 ± 85.7	1082 (%100)
Puncture-to-Recanalization Time (min)		6–182	46.13 ± 23.6	1082 (%100)
Number of Passes		1–15	2 ± 1.2	1082 (%100)
First Pass Recanalization	No			596–55.1%
	Yes			486–44.9%
24th Hour NIHSS		0–32	9.6 ± 7	1082 (%100)
ASPECTS Score		6–10	8.4 ± 1.2	1082 (%100)
Occlusion Location	MCA M1			914–80%
	MCA M2			164–14.3%
	ICA T,L			63–5.5%
	ACA A1			2–0.2%

Min: minimum; Max: maximum; SD: standard deviation; COPD: chronic obstructive pulmonary disease; PLT: platelet; RDW: red cell distribution width; NLR: neutrophil-to-lymphocyte ratio; PLR: platelet-to-lymphocyte ratio; NIHSS: National Institutes of Health Stroke Scale; min: minutes; ASPECTS: Alberta Stroke Program Early CT Score; MCA: middle cerebral artery; ICA: internal carotid artery; ACA: anterior cerebral artery.

**Table 2:** Correlation of inflammatory parameters with admission NIHSS score and 24th hour NIHSS score.

			Neutrophil	Lymphocyte	PLT	NLR	PLR	Admission NIHSS	24th Hour NIHSS
Spearman’s rho	Neutrophil	Correlation Coefficient	1.000	−0.217 **	0.245 **	0.716 **	0.327 **	0.131 **	0.152 **
		*p*		0.000	0.000	0.000	0.000	0.003 *	0.000 *
		*n*	1082	1082	1082	1082	1082	1082	1082
	Lymphocyte	Correlation Coefficient	−0.217 **	1.000	0.162 **	−0.801 **	−0.833 **	−0.061	−0.095
		*p*	0.000		0.000	0.000	0.000	0.482	0.627
		*n*	1082	1082	1082	1082	1082	1082	1082
	PLT	Correlation Coefficient	0.245 **	0.162 **	1.000	0.043	0.353 **	0.004	0.042
		*p*	0.000	0.000		0.159	0.000	0.891	0.170
		*n*	1082	1082	1082	1082	1082	1082	1082
	NLR	Correlation Coefficient	0.716 **	−0.801 **	0.043	1.000	0.766 **	0.143 **	0.186 **
		*p*	0.000	0.000	0.159		0.000	0.018 *	0.005 *
		*n*	1082	1082	1082	1082	1082	1082	1082
	PLR	Correlation Coefficient	0.327 **	−0.833 **	0.353 **	0.766 **	1.000	0.021	0.037
		*p*	0.000	0.000	0.000	0.000		0.498	0.218
		*n*	1082	1082	1082	1082	1082	1082	1082

Correlation coefficients (rho): negligible: 0.00–0.10; weak: 0.10–0.39; moderate: 0.40–0.69; strong: 0.70–0.89. PLT: platelet; NLR: neutrophil-to-lymphocyte ratio; PLR: platelet-to-lymphocyte ratio; NIHSS: National Institutes of Health Stroke Scale. * correlation is significant at the 0.05 level (2-tailed). ** correlation is significant at the 0.01 level (2-tailed).

**Table 3:** Association of demographic, clinical and laboratory data with 90th day clinical outcome.

	mRS 0-1-2				mRS 3-4-5-6			
		Min–Max	Mean ± SD	*n*-%	Min–Max	Mean ± SD	*n*-%	*p* Value
Risk Factors								
Age		19–80	63.4 ± 12.8	636–58.7%	31–80	67.6 ± 10.7	446–41.3%	0.538 ^t^
Gender	Female			321–50.5%			224–50.2%	0.936 ^x2^
	Male			315–49.5%			222–49.8%	
Hypertension	No			274–43.1%			152–34.1%	0.003 * ^x2^
	Yes			362–56.9%			294–65.9%	
Diabetes Mellitus	No			468–73.6%			257–57.6%	0.001 * ^x2^
	Yes			168–26.4%			189–42.4%	
Coronary Artery Disease	No			426–67%			266–59.6%	0.013 * ^x2^
	Yes			210–33%			180–40.4%	
Asthma and/or COPD	No			582–91.5%			382–85.7%	0.002 * ^x2^
	Yes			54–8.5%			64–14.3%	
Atrial Fibrillation	No			382–60.1%			258–57.8%	0.466 ^x2^
	Yes			254–39.9%			188–42.2%	
Alcohol Consumption	No			588–92.5%			408–91.5%	0.56 ^x2^
	Yes			48–7.5%			38–8.5%	
Smoker	No			453–71.2%			335–75.1%	0.157 ^x2^
	Yes			183–28.8%			111–24.9%	
Previous Stroke History	No			561–88.2%			373–83.6%	0.031 * ^x2^
	Yes			75–11.8%			73–16.4%	
Hyperlipidemia	No			278–43.7%			237–53.1%	0.002 * ^x2^
	Yes			358–56.3%			209–46.9%	
Inflammatory Parameters								
Neutrophil (10⁹/L)		0.1–90.8	7 ± 5.08	636	0.6–112.18	9.5 ± 11.8	446	0.001 * ^t^
Lymphocyte (10⁹/L)		0.2–51.7	2.19 ± 2.7	636	0.36–20.5	2.3 ± 2.5	446	0.956 ^t^
PLT (10⁹/L)		10–996	236.6 ± 88.2	636	10–996	243.1 ± 97.8	446	0.104 ^t^
RDW (%)		9.5–172	14.8 ± 5.8	636	9.9–53.9	14.9 ± 3.89	446	0.03 * ^t^
NLR		0.02–124.45	4.9 ± 6.3	636	0.13–56.37	5.98 ± 6.2	446	0.013 * ^t^
PLR		4.74–1930	152.4 ± 123.2	636	3.33–1350	160.9 ± 123.9	446	0.318 ^t^
Admission NIHSS		2–27	13.5 ± 4.5	636	5–32	16.5 ± 4.6	446	0.001 * ^t^
Symptom-to-Inguinal Puncture Time (min)		15–360	185.5 ± 83.8	636	9–360	205.4 ± 87	446	0.001 * ^t^
Puncture-to-Recanalization Time (min)		6–172	43.2 ± 21.8	636	7–182	50.1 ± 25.4	446	0.001 * ^t^
Number of Passes		1–15	1.9 ± 1.2	636	1–8	2.1 ± 1.2	446	0.001 * ^t^
First Pass Recanalization	No			325–51.1%			271–60.8%	0.002 * ^x2^
	Yes			311–48.9%			175–39.2%	
24th Hour NIHSS		0–32	6.4 ± 5.3	636	0–32	14.3 ± 6.6	446	0.001 * ^t^

* *p*-values based on chi-square test for categorical variables and *t*-test for continuous variables. mRS: modified Rankin Scale; Min: minimum; Max: maximum; SD: standard deviation; COPD: chronic obstructive pulmonary disease; PLT: platelet; RDW: red cell distribution width; NLR: neutrophil-to-lymphocyte ratio; PLR: platelet-to-lymphocyte ratio; min: minutes; NIHSS: National Institutes of Health Stroke Scale; t: *t*-test; ^x2^: chi-square test.

**Table 4:** Univariate and multivariate logistic regression analysis.

	Univariate Analysis Results		Multivariate Analysis Results	
	OR (95%CI)	*p*	aOR (95%CI)a	aP
Neutrophil, ×10^9^/L	1.000 (0.999–1.001)	0.649	0.988 (0.962–1.014)	0.630
Lymphocyte, ×10^9^/L	1.032 (0.990–1.075)	0.134	1.000 (0.959–1.044)	0.096
NLR	1.000 (0.998–1.001)	0.594	1.027 (0.989–1.066)	0.557
PLR	1.001 (1.000–1.002)	0.273	1.002 (0.998–1.006)	0.268
RDW	1.007 (0.985–1.028)	0.539	0.996 (0.974–1.020)	0.527
24th Hour NIHSS	1.242 (1.209–1.277)	0.000	0.831 (0.803–0.859)	0.000
Puncture–Recanalization Time	1.013 (1.007–1.018)	0.000	0.981 (0.973–0.989	0.000
First Pass Recanalization	0.675 (0.528–0.863	0.002	1.130 (0.723–1.766)	0.591

OR: odds ratio; aOR: adjusted odds ratio; aP: adjusted *p* value; CI: confidence interval, adjusted for age, sex, diabetes mellitus, coronary heart disease, prior stroke; NLR: neutrophil-to-lymphocyte ratio; PLR: platelet-to-lymphocyte ratio; RDW: red cell distribution width; NIHSS: National Institutes of Health Stroke Scale.

**Table 5:** ROC analysis for 24th hour NIHSS score and puncture-to-recanalization time predicting poor 90-day outcomes.

Variables	AUC	95%CI	*p*	Cut-Off	Sensitivity	Specificity
24th hour NIHSS	0.839	0.815–0.863	0.00	8.50	0.812	0.753
Puncture-to-recanalization time (min)	0.578	0.543–0.613	0.00	43.5	0.543	0.583

AUC > 0.60 is weak; AUC > 0.70 is acceptable; 0.80–0.90 is excellent; and greater than 0.90 is outstanding. Therefore, variables with an AUC value above 0.60 were analyzed. AUC: area under curve; CI: confidence interval; NIHSS: National Institutes of Health Stroke Scale; min: minutes.

## Data Availability

Due to the nature of the research, for ethical reasons supporting data is not available.

## Supplementary Materials

The following supporting information can be downloaded at: https://www.mdpi.com/article/10.3390/diagnostics14242880/s1, Figure S1: NLR box Plot in terms of mRS; Table S1: Association of Demographic, Clinical and Laboratory data with 90th Day Clinical Outcome; Table S2: Multivariate Regression Analysis Results.

## Abbreviations

NLR	Neutrophil-to-Lymphocyte Ratio
mRS	Modified Rankin Scale
NIHSS	National Institutes of Health Stroke Scale
OR	Odds ratio
CI	Confidence Interval
NETs	Neutrophil extracellular traps
AIS	Acute Ischemic Stroke
BBB	Blood Brain Barrier
HT	Hemorrhagic Transformation
ICA	Internal Carotid Artery
mTICI	Modified treatment in cerebral infarction
AF	Atrial Fibrilation
HL	Hyperlipidemia
CHD	Coronary Heart Disease
BP	Blood Pressure
ASPECTS	Alberta Stroke Program Early Computed Tomography score
BG	Blood Glucose
RDW	Red Blood Cell Distribution Width
PLT	Platelet
PLR	Platelet/Lymphocyte Ratio
SD	Standard Deviation
COPD	Chronic Obstructive Pulmonary Disease
Min	Minimum
Max	Maximum
min	Minutes
MCA	Middle Cerebral Artery
ACA	Anterior Cerebral Artery
aOR	Adjusted Odds Ratio
aP	Adjusted P Value
ROC	Receiver Operating Characteristic
AUC	Area Under curve
MT	Mechanical Thrombectomy
