# Supplementary material for: Impact of Neutrophil-to-Lymphocyte Ratio on Stroke Severity and Clinical Outcome in Anterior Circulation Large Vessel Occlusion Stroke
Source: Diagnostics (Basel). 2024 Dec 21;14(24):2880. doi: 10.3390/diagnostics14242880 (PMC11675451; doi:10.3390/diagnostics14242880)
Supplement: Supplementary file 1 [file diagnostics-14-02880-s001.zip › diagnostics-3372362-supplementary.pdf]

		**Supp Table S1: Association of Demographic, Clinical and Laboratory data with 90th Day Clinical Outcome**								
		mRS 0-1-2					mRS 3-4-5-6			
		Min-Max	Mean ± SD	25% – Median – 75%	n - %	Min-Max	Mean ± SD	25% – Median – 75%	n - %	*P* Value
Risk Factors										
Age		19–80	63.4 ± 12.8		636–58.7%	31–80	67.6 ± 10.7		446–41.3%	0.538^{t}
Gender	Female				321–50.5%				224 – 50.2%	
	Male				315–49.5%				222–49.8%	0.936^{x2}
Hypertension	No				274–43.1%				152–34.1%	
	Yes				362–56.9%				294–65.9%	**0.003*** ^{x2}
Diabetes Mellitus	No				468–73.6%				257–57.6%	
	Yes				168–26.4%				189 – 42.4%	**0.001*** ^{x2}
Coronary Artery Disease	No				426–67%				266–59.6%	
	Yes				210–33%				180–40.4%	**0.013*** ^{x2}
Asthma and/or COPD	No				582–91.5%				382–85.7%	
	Yes				54–8.5%				64–14.3%	**0.002*** ^{x2}
Atrial Fibrilation	No				382–60.1%				258–57.8%	
	Yes				254–39.9%				188–42.2%	0.466 ^{x2}
Alcohol consumption	No				588–92.5%				408–91.5%	
	Yes				48–7.5%				38–8.5%	0.56 ^{x2}
Smoker	No				453–71.2%				335–75.1%	
	Yes				183–28.8%				111–24.9%	0.157 ^{x2}
Previous Stroke History	No				561–88.2%				373–83.6%	
	Yes				75–11.8%				73–16.4%	**0.031*** ^{x2}
Hyperlipidemia	No				278–43.7%				237–53.1%	**0.002*** ^{x2}

	Yes				358–56.3%				209–46.9%	
Inflammatory Parameters										
Neutrophil (10^9 /L)		0.1–90.8	7 ± 5.08	4.45–6.09–8.38	636	0.6–112.18	9.5 ± 11.8	4.87–7.00–10.55	446	**0.001*** ^{t}
Lymphocyte (10^9 /L)		0.2–51.7	2.19 ± 2.7	1.21–1.75–2.59	636	0.36–20.5	2.3 ± 2.5	1.14–1.71–2.60	446	0.956^{t}
PLT (10^9 /L)		10–996	236.6 ± 88.2	183.25–220.50–269.75	636	10–996	243.1 ± 97.8	183.00–226.00–280.00	446	0.104^{t}
RDW (%)		9.5–172	14.8 ± 5.8	13.00–13.80–15.20	636	9.9–53.9	14.9 ± 3.89	13.20–14.00–15.30	446	**0.03*** ^{t}
NLR		0.02–124.45	4.9 ± 6.3	1.97–3.26–6.23	636	0.13–56.37	5.98 ± 6.2	2.06–3.96–7.95	446	**0.013*** ^{t}
PLR		4.74–1930	152.4 ± 123.2	83.07–126.12–185.97	636	3.33–1350	160.9 ± 123.9	83.53–133.09–199.52	446	0.318^{t}
Admission NIHSS		2–27	13.5 ± 4.5		636	5–32	16.5 ± 4.6		446	**0.001*** ^{t}
Symptom-to- inguinal puncture time (min)		15–360	185.5 ± 83.8		636	9–360	205.4 ± 87		446	**0.001*** ^{t}
Puncture to Recanalization Time (min)		6–172	43.2 ± 21.8		636	7–182	50.1 ± 25.4		446	**0.001*** ^{t}
Number of Pass		1–15	1.9 ± 1.2		636	1–8	2.1 ± 1.2		446	**0.001*** ^{t}
First Pass recanalization	No				325–51.1%				271–60.8%	
	Yes				311–48.9%				175–39.2%	**0.002*** ^{x2}
24th hour NIHSS		0–32	6.4 ± 5.3		636	0–32	14.3 ± 6.6		446	**0.001*** ^{t}

*p-values based on chi-square test for categorical variables and t-test for continuous variables.

Supp: Supplementary, mRS: Modified Rankin Scale, Min: Minimum, Max: Maximum, SD: Standard Deviation, COPD: Chronic obstructive pulmonary disease, PLT: Platelet, RDW: Red cell distribution width, NLR: Neutrophil to lymphocyte ratio, PLR: Platelet to lymphocyte ratio, min: minutes, NIHSS: National Institutes of Health Stroke Scale, t: t-test, x2: chi-square test

Supp Table S2: Multivariate Regression Analysis Results

	B	S.E	p	aOR (95%CI)a	*aP*
Neutrophil	-0.005	0.004	0.232	0.988(0.962–1.014)	.630
Lymphocyte.	0.021	0.022	0.329	1.000(0.959–1.044)	.096

NLR	0.108	0.068	0.112	1.027(0.989–1.066)	.557
PLR	-0.002	0.002	0.318	1.002(0.998–1.006)	.268
RDW	-0.004	0.012	0.757	0.996(0.974–1.020)	.527
24th hour NIHSS	-0.195	0.019	**0.000***	0.831(0.803–0.859)	**.000***
Puncture Recanalization Time	-0.020	0.005	**0.000***	0.981(0.973–0.989	**.000***
First Pass rekanalizasyon	-0.021	0.291	0.942	1.130(0.723–1.766)	.591

Supp: Supplementary, OR, odds ratio; aOR, adjusted odds ratio; aP, adjusted p value; CI, confidence interval; adjustment for age, sex, diabetes mellitus, coronary heart disease, prior stroke, NLR: Neutrophil to lymphocyte ratio, PLR: Platelet to lymphocyte ratio, RDW: Red cell distribution width, NIHSS: National Institutes of Health Stroke Scale

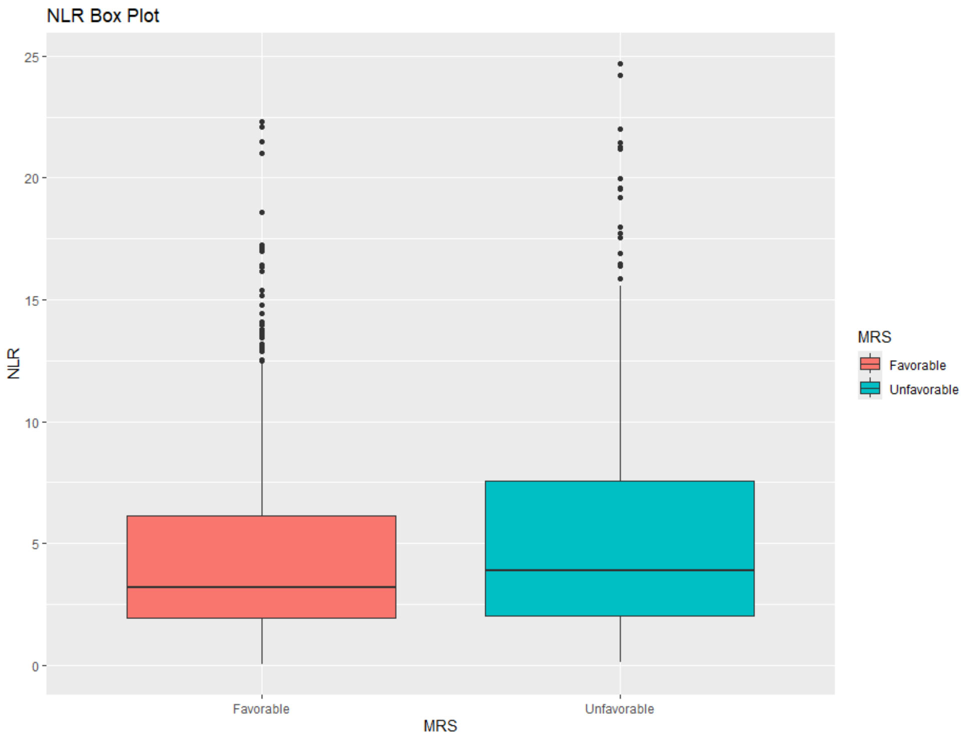

Supp Figure S1: NLR box Plot in terms of mRS

Supp: Supplementary, NLR: Neutrophil to lymphocyte ratio, mRS: Modified Rankin Scale
